# Prevalence and determinants of malaria infection among children of local farmers in Central Malawi

**DOI:** 10.1186/s12936-020-03382-7

**Published:** 2020-08-27

**Authors:** Emmanuel Chilanga, Delphine Collin-Vézina, Heather MacIntosh, Claudia Mitchell, Katrina Cherney

**Affiliations:** 1 University of Livingstonia, Livingstonia, Malawi; 2 School of Social Work, McGill University, Montreal, Canada; 3 Faculty of Education, McGill University, Montreal, Canada

**Keywords:** Malaria infection, Prevalence, Risk factors, Children under five, Rural Malawi

## Abstract

**Background:**

Malaria is a leading cause of morbidity and mortality among children under 5 years in Malawi, and especially among those from rural areas of central Malawi. The goal of this study was to examine the prevalence and determinants of malaria infection among children in rural areas of Dowa district in central Malawi.

**Methods:**

A multistage, cross-sectional study design was used to systematically sample 523 child-mother dyads from postnatal clinics. A survey was administered to mothers and a rapid malaria infection diagnostic test was administered to children. The main outcome was positive malaria diagnostic tests in children. Logistic regressions were used to determine risk factors associated with malaria among children aged 2 to 59 months.

**Results:**

The prevalence of malaria among children under 5 years was 35.4%. Results suggest that children of mothers who experienced recent intimate partner violence (IPV) were more likely to be diagnosed with malaria (AOR: 1.88, 95% CI 1.19–2.97; *P* = 0.007) than children of mothers who did not. Children of mothers who had no formal education were more likely to be diagnosed with malaria (AOR: 2.77, 95% CI 1.24–6.19; *P* = 0.013) than children of mothers who had received secondary education. Children aged 2 to 5 months and 6 to 11 months were less likely to be diagnosed with malaria (AOR: 0.21, 95% CI 0.10–0.46; *P* = 0.000 and AOR: 0.43; 95% CI 0.22–0.85; *P* = 0.016, respectively) than children aged 24 to 59 months.

**Conclusion:**

The prevalence of malaria infection among children in the study area was comparable to the national level. In addition to available malaria control programmes, further attention should be paid to children whose mothers have no formal education, children aged 24 to 59 months, and children of mothers that are exposed to IPV in the area.

## Background

Malaria is a mosquito-borne disease that kills a significant number of people in Africa every year [1]. The pathology is mainly caused by the *Plasmodium falciparum* parasite and is transmitted to human beings through female *Anopheles* mosquito bites [2]. In 2017, 61% of cases of malaria worldwide were in children under the age of 5 years. Geographically, approximately 92% (200 million) of malaria cases in the world were diagnosed in Africa, claiming about 404,550 lives [1].

In Malawi, malaria is among the three most significant public health issues. Nearly 4 million people are diagnosed with the infection every year [3]. Malawi accounts for 2% of malaria cases worldwide and is among the top 15 countries with a high malaria burden [1]. Children under 5 years and pregnant women are at a high risk for malaria morbidity compared to other groups in Malawi [4]. Since 2005, the Malawi Government has been implementing comprehensive malaria control programmes that target more than 85% of its population. The two main strategies have been preventing the malaria vector mosquitoes from biting people, and case management. Prevention efforts include promoting the use of insecticide-treated nets (ITNs) and indoor spraying of insecticide. Case management includes diagnostic testing and prescribing anti-malarial drugs to children with positive malaria tests [5]. These strategies are combined with messages about social behavioural changes in order to increase community uptake and utilization [6].

Despite these investments, little progress has been made so far to reduce the burden of malaria in children under five in Malawi. Studies have shown that the prevalence of malaria among children detected by a gold standard microscopy technique was at 28% in 2012; it increased to 33% in 2014 and slightly dropped to 24% in 2017 [7]. Malaria morbidity among children is not evenly distributed across Malawi. According to national data collected through malaria rapid diagnostic tests (RDTs) and microscopy in 2017, the prevalence of child malaria is significantly higher in rural areas (40.6 and 27.5%) compared to urban areas (6 and 4%), respectively. In addition, the prevalence of malaria among children in central Malawi was higher (39.7%) compared to children in southern (36.4%) and northern (19.4%) regions [8]. These studies suggest that geography plays a significant role in malaria prevalence among children. Therefore, there is a need to broaden the scope of studies that consider social and environmental risk factors for malaria to inform local policies and programmes.

The study was conducted in rural areas of Dowa district in Malawi because of two reasons. First, Dowa district is one of the highest-risk areas for child malaria in Malawi. Despite this, there is no literature to date that specifically focuses on this region apart from national aggregated studies [8]. Second, the research aimed at contributing to the study by Sassi [9], who assessed the risk factors of child under-nutrition in the Dowa district. In the study, the researcher used household access to mosquito nets as a proxy variable for malaria control among children. The assumption was that children from households without mosquito nets would be more likely to suffer from malaria as there are synergistic interactions between the two child morbidities. The present study specifically used cross-sectional quantitative data to examine individual and household risk factors for child malaria infection in order to understand the phenomenon in the Dowa district.

## Methods

### Study setting

**Fig. 1:**
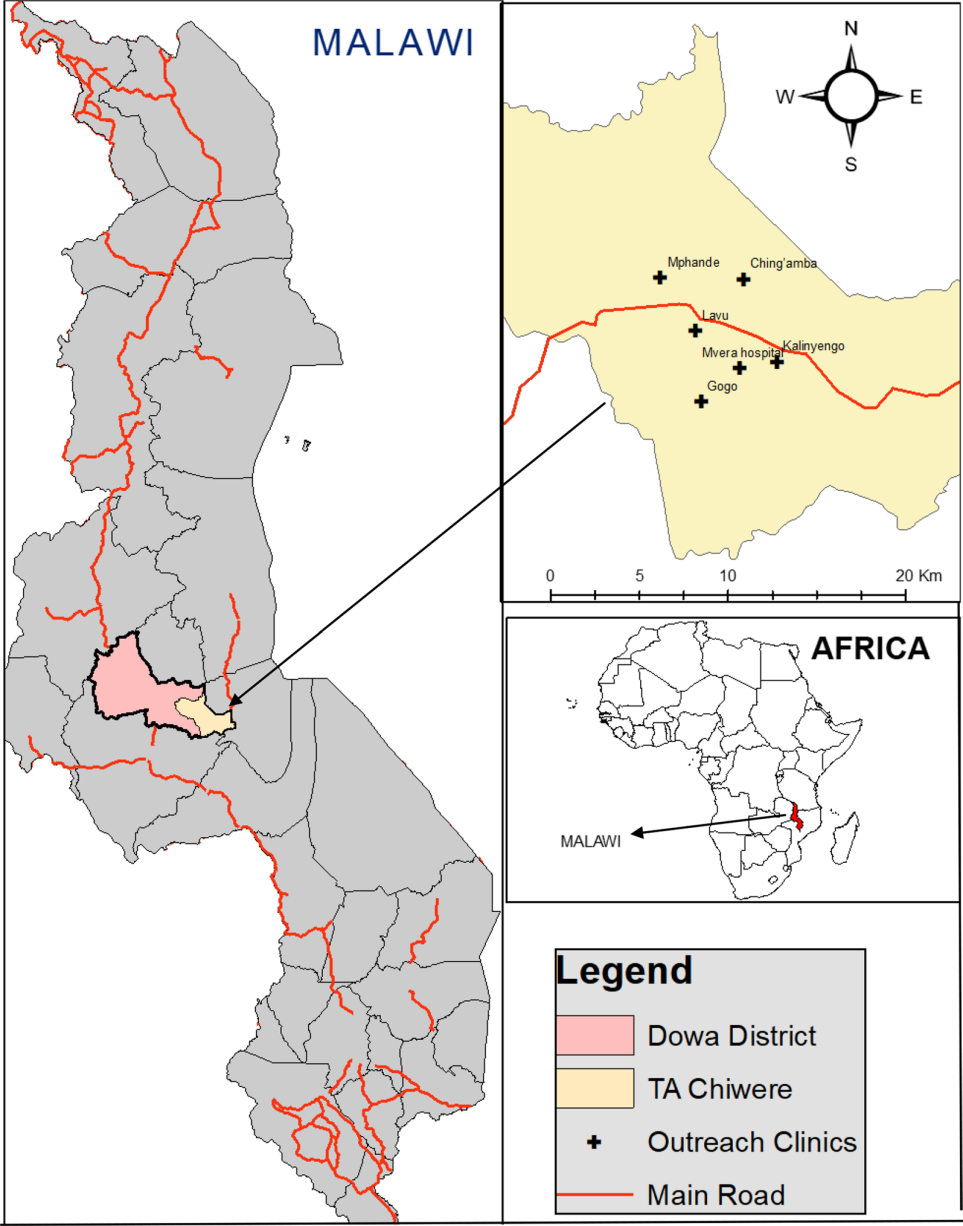
The research setting

The study was implemented in six postnatal clinics in the Dowa district of central Malawi in southern Africa (Fig. 1). Malawi has a population of about 17,563,749 people [8]. In 2017, about 71% of the population was living in extreme poverty according to United Nations indicators [10]. Malaria is an endemic disease in Dowa and surrounding districts, but higher numbers of cases are recorded during and after the rainy season (December to July) due to increased potential breeding environments [9, 11]. This study was conducted between May and September 2018 because this period would allow to capturing of average malaria cases in the study areas.

### Study sample

A multistage, descriptive, cross-sectional study design was employed to select a representative sample of children aged 2 to 59 months and their mothers in the Dowa district. A random selection of 6 out of 8 outreach clinics that were part of the Mvera mission hospital was conducted. The selected clinics were located in Gogo, Ching’amba, Mkhalanjoka, Kalinyengo, Mvera and Mphande, within approximately 5–10 km of the Mvera mission hospital. During the study, the Mvera mission hospital served a population of 27,719 people, of whom 5240 were mothers with a child under 5 years old. A total population of 4527 mothers with children between 2 and 59 months were identified in postnatal registers in the 6 randomly selected postnatal clinics. The sample size was determined by a Raosoft sample size calculator [12]. A margin of error of 5% with a 95% confidence level and 50% response distribution was set. A systematic sampling strategy was used to select a sample of 523 children and their mothers from the postnatal registers. The first child-mother dyad was randomly picked and subsequently every 9th child-mother dyad were selected.

### Participant recruitment

Selected mothers and their children were contacted at the 6 postnatal clinics during the regular monthly health screening for children. The screening programme is an initiative of the Malawi Government to promote maternal and child health through a framework of a continuum of care for mothers, newborns and children [13]. Community health workers who were assigned as research assistants sought informed consent from mothers to take part in the study. All interviews took place in the consultation room at each outreach postnatal clinic using a pre-tested questionnaire. The questionnaire contained questions on household socio-demographic characteristics, household food security, house structure type, use of mosquito nets, and maternal exposure to intimate partner violence (IPV). The child’s and the mother’s anthropometric and health status data from their health passports were recorded in the questionnaire after their health screening programme was completed [14].

## Measures

### Outcome variable

The main outcome variable of the study was malaria infection in children 2 to 59 months old. The term child malaria infection was operationalized as the presence of the malaria parasite in children’s red blood cells as recorded in the child’s health passport [15]. In all the postnatal clinics, RDTs were used to assess the malaria parasitaemia in children. If the diagnostic test was positive, the child was coded as 1 = malaria infection, and 0 = otherwise.

#### Explanatory variables

The selection of potential covariates of child malaria infection in the regression models was based on current literature in Malawi and other countries in sub-Saharan Africa (SSA) [4, 16–20]. These were characteristics of the children, characteristics of the parents that influence childcare practices and characteristics of the household. The covariate variables were child gender, age and weight at birth. Variables that captured child’s history of other morbidities in the past 30 days as reported by the mother were also included. These included diarrhoeal episodes and acute respiratory infection (ARI); child de-worming in the past 12 months was also considered. Child nutrition status was determined through height-for-age, weight-for-height and weight-for-age Z-score values. Child stunting, underweight and wasting were categorized as those that were ≤ -2 standard deviations of height–for-age, weight-for-age and weight-for-height Z-scores [21].

Other independent variables that were considered as risk factors for child malaria included the mother’s age, level of education, pregnancy planning and exposure to IPV perpetrated by their current or recent husband. Cases of IPV was assessed by using a WHO multi-country study questionnaire on women’s health and life experiences that was validated and used in Malawi [22, 23]. The questionnaire contained 18 items that made up four sub-scales measuring different forms of IPV: physical abuse, emotional abuse, controlling behaviour, and sexual abuse. Maternal exposure to IPV was operationalized as any mother who reported that they experienced any form of IPV.

Fathers’ characteristics were also considered as risk factors. These included level of education, age and health risk behaviours, such as alcohol consumption and smoking. Household malaria predisposing and enabling factors in Malawi such as the use of an ITN, household poverty, type of dwelling, and presence of animals in the house were included. Mothers were asked if their child had an ITN, and whether the child slept under the net the night before the survey. Household poverty was defined based on the international poverty measure of US$1.90 a day [24]. The presence of animal kraals/sheds within 1–10 m of the dwelling house was considered a risk factor [25]. Mothers were also asked if their houses had been sprayed with insecticides and how many people were sleeping in the house.

Survey enumerators administered the survey on android tablets using an Open Data Kit (ODK). A WHO protocol for conducting research on sensitive topics was adopted because some of the questions in the study focused on domestic violence [26, 27]. Enumerator orientation and questionnaire pre-testing was conducted for 5 days. A PhD candidate in social work, a clinical officer and an environmental health officer were responsible for training the enumerators. The research team, including the enumerators, had professional training in community health, nutrition and primary health care.

### Research ethics review

Ethics approval to conduct this study was obtained from the University of Livingstonia research ethics committee in Malawi (protocol number: UNILIA-REC-4/18) and the Research Ethics Board of McGill University in Canada (protocol number: REB File #: 503-0518). Written permission was also sought from the Dowa district commissioner’s office, the Dowa district health office and the Mvera mission hospital management. Oral consent was obtained from local health leaders and research participants in the study areas.

### Data analysis

The Kolmogorov–Smirnov test was used to test the normality of the distribution of numerical variables. These include age, number of children, number of household members, and household food security. Categorical variables were constructed from the numerical data because the data were not normally distributed [28]. Bivariate logistic regressions were performed to examine significant predictors of child malaria. Significant predictors of child malaria at the bivariate level of (p  < 0.05) were included in the final multivariable logistic regression model using the forward enter method.

Multicollinearity of explanatory variables were tested and a variance inflation factor (VIF) of 5143, was obtained which indicated independence among the explanatory variables both at the individual and the cluster level. Consequently, a fixed effects model was used to account for the clustering effect in the analysis. The results of the multivariable analysis have been reported as crude and adjusted odds ratios (AOR) with a 95% confidence interval (CI). A *p* value of less than 0.05 was considered statistically significant in the study. The data were analysed using an IBM Statistical Package of Social Sciences (SPSS) for Windows version 23.0 (IBM Corp., Armonk, NY, USA).

## Results

### Socio-demographic characteristics of study population

Socio-demographic and malaria infection data for all 523 selected children aged 2 to 59 months were obtained over 4 months (see Table 1). In terms of gender, 49.1% of the children were girls and 50.9% were boys. In terms of age, 13.4% of the sample was aged 2 to 5 months, 17.2% was aged 6 to 11 months, 29.6% was aged 12 to 23 months, and 41.9% was aged 24 to 59 months. It was observed that 14.3% of the selected children were born with a low birth weight (birth weight of less than 2.5 kg); 27.2% of the mothers reported that their children did not sleep under mosquito nets the night before the survey; 67.1% of mothers reported that their children had signs of fever 30 days preceding the survey. The study found that 42.0% of the children were stunted, and 11.3% were underweight.

**Table 1:** Characteristics of children, mothers, fathers, and the environment

Mothers’ characteristics		
Age (years)	N = 523	%
15–19	39	7.5
20–29	299	57.3
30–39	155	29.6
40–49	29	5.5
Education		
No education	80	15.3
Primary	356	68.1
Secondary	87	16.6
Received childcare education		
Yes	464	88.7
No	59	11.3
Exposed to IPV		
Yes	392	75.0
No	131	25
Confidant		
Yes	382	73.0
No	141	27.0
Children’s characteristics		
Nutrition status		
Stunted	219	42.0
Not stunted	304	58.0
Underweight	59	11.3
Normal weight	57	10.6
Fever		
No	172	32.9
Yes	351	67.1
Malaria		
No	338	62.6
Yes	185	35.4
Cough		
Yes	213	40.7
No	308	58.9
Child birth weight		
Normal	447	85.6
Low birth weight	75	14.3
De-wormed		
Yes	240	45.9
No	283	54.1
Sleep under mosquito net		
Yes	380	72.8
No	142	27.2
Gender		
Female	257	49.1
Male	275	50.9
Age (months)		
2–5	59	13.4
6–11	90	17.2
12–23	155	29.6
24–59	219	41.9
Husbands’ characteristics		
Age category		
15–24	85	16.3
25–34	239	45.7
35–49	199	38.0
Educational level		
No education	76	14.5
Primary	296	56.2
Secondary	150	28.7
Household characteristics		
Poverty level (US$1.90/day)		
Below poverty line	500	95.6
Above poverty line	23	4.4
Keep pigs around		
Yes	59	11.3
No	464	88.7
Keep goats around		
Yes	117	22.4
No	406	77.6
Number of children		
1–2	283	54.4
3–4	162	31.0
5 and more	75	14.4
Walls of house		
Mud/sticks	387	74.1
Bricks	135	25.8
Roof of house		
Grass thatched	455	87.0
Iron sheets	67	12.8
Indoor residual spraying (IRS)	0	0

In terms of parental characteristics, the study found that 15.3% of the mothers had no formal education, 68.1% had a primary education and 16.6% had a secondary education. Seventy-five per cent (n = 392) of the mothers reported that they experienced IPV perpetrated by their current or most recent partner in the past 12 months. Regarding age, 7.5% of the mothers were 15 to 19 years old, 57.3% were 30 to 39 years old, 29.6% were 30 to 39 years old, and 5.5% were 40 to 49 years old. The results show that 88.7% (n = 464) of the mothers reported that they had received childcare counselling during their pregnancy. With respect to the fathers, the study found that 14.5% (n = 76) had no formal education, 56.2% (n = 296) had a primary education, and 28.7% (n = 150) had a secondary education. Slightly fewer than half of the husbands (43%) were beer drinkers and a quarter (26%) were tobacco smokers.

The study found that 11.3% (n = 59) of the households had pigs and 22.4% (n = 117) had goats in kraals/sheds close to their dwelling. Regarding house construction materials, 25.8% (n = 135) of the children lived in brick-walled houses, and only 12.8% (n = 67) lived in iron-roofed houses.

### Prevalence and factors associated with child malaria in bivariate and multivariate analyses

The study found that 35% of children (n = 185) were diagnosed with the malaria parasite within 48 h prior to the research interview. There was no gender difference in malaria cases among the sampled children (*χ2* = 0.00, *df* = 1, *p *= 0.987). Unadjusted logistic regressions (Table 2) indicated that children of mothers who had no formal education were more likely to be diagnosed with the malaria parasite than children of mothers with a secondary school education (crude odds ratio (COR): 2.92, 95% CI 1.44–5.91, *P* = 0.003). Children who were in the age range of 2 to 5 and 6 to 11 months were less likely to be diagnosed with malaria compared to children who were in the age range of 24 to 59 months (COR: 0.14, 95% CI 0.07–0.26, *P* = 0.000 and COR: 0.26, 95% CI 0.16–0.44, *P* = 0.000, respectively). Children whose mothers experienced IPV in the form of controlling behaviour in the past 12 months were more likely to be diagnosed with the malaria parasite than children whose mother did not (*COR*: 2.92, 95% CI 1.44–5.91, *P* = 0.003). Children who were consistently sleeping under mosquito nets were less likely to be diagnosed with the malaria parasite than children who were not regularly sleeping under the net (COR: 0.45, 95% CI 0.30–0.75, *P* = 0.001). Children who did not receive de-worming drugs were more likely to be diagnosed with the malaria parasite than children who were de-wormed (COR: 2.61, 95% CI 1.79–3.82, *P* = 0.000). Children whose mothers had female confidants were less likely to be diagnosed with the malaria parasite than children whose mothers had no confidant (COR: 0.64, 95% CI 0.43–0.99, *P* = 0.048). Children whose fathers were in the age range of 15 to 24 years old were less likely to suffer from malaria than children whose fathers were in the age range of 35 to 49 (COR: 0.55, 95% CI 0.33–0.91, *P* = 0.021). Finally, children whose mothers were in the age range of 30 to 39 years old were less likely to suffer from malaria than children whose mothers were 40 to 49 years old (COR: 0.14, 95% CI 0.07–0.26, *P* = 0.000).

**Table 2:** Crude and adjusted odds ratios (95% CI) for factors associated with child malaria in the Dowa district

Variables		Crude OR	(95% CI)	*P* value	Adjusted OR	(95% CI)	*P*-value
Mother’s education							
No education		2.92	1.44–5.91	0.003	2.77	1.24–6.19	0.013
Primary		1.12	0.69–1.80	0.656	1.07	0.62–1.87	0.806
Secondary		1			1		
Child’s age (months)							
2–5		0.14	0.07–0.26	0.000	0.21	0.10–0.46	0.000
6–11		0.26	0.16–0.44	0.000	0.43	0.22–0.85	0.016
12–23		0.64	0.40–1.02	0.063	0.91	0.52–1.57	0.136
24–59		1			1		
Child de-wormed							
No		2.61	1.79–3.82	0.000	1.42	0.84–2.39	0.191
Yes		1			1		
Child’s ITN use							
Yes		0.47	0.30–0.75	0.001	0.72	0.43–1.20	0.200
No		1			1		
Husband’s age							
15–24		0.55	0.33–0.91	0.021	0.83	0.47–1.54	0.588
25–34		1.09	0.73–1.63	0.678	1.24	0.78–1.96	0.362
35–49		1			1		
IPVAM (control)							
Yes		1.83	1.22–2.74	0.003	1.88	1.19–2.97	0.007
No							
Confidant							
Yes		0.64	0.43–0.99	0.48	0.70	0.43–1.12	0.136
No		1					
Mother’s age							
15–19		0.37	0.12–1.19	0.096	0.39	0.11–1.48	0.168
20–29		0.40	0.13–1.07	0.068	0.45	0.15–1.34	0.152
30–39		0.31	0.11–0.86	0.025	0.29	0.10–0.90	0.032
40–49		1					

1 is a reference category

In multivariable analysis (Table 2), the odds of children being diagnosed with malaria was higher among children whose mothers had no formal education than among children whose mothers had a secondary education (AOR: 2.77, 95% CI 1.24–6.19, *P* = 0.013). It was also found that children whose mothers had experienced IPV in the form of controlling behaviour in the past 12 months had higher odds of being diagnosed with the malaria parasite compared to children whose mothers did not experience IPV in the past year (AOR: 1.88, 95% CI 1.19–2.97, *P* = 0.007). Children who were 2 to 5 and 6 to 11 months old were less likely to suffer from malaria than children who were 24 to 59 months old (AOR: 0.21, 95% CI 0.10–0.46, *P* = 0.000 and AOR: 0.43, 95% CI 0.22–0.85, *P* = 0.016, respectively). Finally, children of mothers who were 30 to 39 years old were less likely to be diagnosed with the malaria parasite than children whose mothers were 40 to 49 years old (AOR: 0.29, 95% CI 0.10–0.90, *P* = 0.032).

## Discussion

This study examined the prevalence of and risk factors for malaria infection among children 2 to 59 months old in order to contribute to the understanding of various socio-demographic determinants associated with poor child health in rural areas of the Dowa district in Malawi. The prevalence of child malaria in this study area was 35.4%, which was equivalent to the national malaria prevalence in 2017 (36%) [3] and just slightly lower than malaria prevalence in central and rural Malawi, at 39.7 and 40.6%, respectively. This finding raises concerns regarding whether long-lasting insecticidal nets (LLINs) and sulfadoxine-pyrimethamine preventive treatment programmes, that were introduced in the study area in 2007 and 2006, respectively and were still running in 2019, are associated with the reduction in malaria prevalence. But a study by Mwendera et al. [29] observed that malaria reduction programmes in Malawi are facing various challenges, including the failure to understand the social cultural context in the uptake of the malaria control programmes. Another study in Malawi suggested that a limited number of health workers and poor prescription of anti-malarial drugs were some of the challenges that constrain malaria prevention in Malawi [30]. In the preceding paragraphs, the study identified risk factors that were significantly associated with child malaria infection in the study areas.

This study found that mothers’ exposure to IPV in the form of controlling behaviour was a significant determinant of malaria infection in children under 5 years of age. The current study supports the findings of a study in South Asia where IPV against women was found to be a predisposing factor for child cough, malaria and diarrhoea [31]. In Tanzania, a nationally representative study also found that children of mothers who were exposed to any form of IPV were at high risk of suffering from fever, cough and diarrhoea [32]. Two explanations can be offered for the observed association between controlling behaviour IPV and child malaria. First, it was anticipate that husbands’ controlling behaviour constrained mothers’ capacity to implement preventative measures suggested by childcare counsellors, including regularly sleeping under the mosquito net. It was also speculated that mothers who were experiencing IPV were more likely to be depressed, which may have compromised their capacity to take care of children [33].

In addition, this study found that children of mothers who had no formal education were more likely to suffer from malaria compared to children whose mothers had a secondary education. This reflects findings from a regional study in SSA, which found that households where children of mothers with a sixth-grade education or higher had lower odds of suffering from malaria (OR = 0.73) compared to children of mothers with lower levels of education [34]. The finding can be explained by a study in Malawi, which found that mothers with higher levels of education were more knowledgeable about malaria prevention and signs, and were therefore more proactive and reactive with regard to prevention than mothers with lower levels of education [35].

Furthermore, this study found that children who were over 2 years old had higher odds of being diagnosed with malaria infection than younger children. This is consistent with other studies which reported that malaria prevalence increases with child age [4, 36]. This may be because younger children in Malawi share a bed with their mothers and are more likely to be covered properly with a blanket or mosquito net than older children. This suggestion is supported by studies in Uganda and other parts of Africa where children who were sharing their mother’s bed were more likely to sleep under a mosquito net compared to children who were not sharing a bed with their mother [37, 38]. Another explanation is that the majority of children in Malawi are weaned from breastfeeding at the age of 2, after which they receive less caregiver attention and have an increased risk of exposure to malaria vectors [39].

Finally, the current study found that children of mothers between 30 to 39 years old were less likely to be diagnosed with malaria than children of mothers between 40 to 49 years old. This finding was surprising, as it was initially assumed that children born from adolescent mothers (16–19 years) would be at higher risk of malaria diagnosis than children born of mothers 40 to 49 years. This suggests that children of both adolescent mothers and older mothers may be at risk of poor health outcomes. This support findings from earlier studies. A longitudinal study in South Africa observed similar prevalence of low birth weight among children of adolescent and adult mothers (aged [40]), and a study in Kenya found that the survival rate of children of adolescent mothers was similar to that of children of older mothers (aged [41]). Therefore, a suggestion is made that child malaria mitigation programmes in Dowa district should also pay attention to the needs of older mothers.

### Implications for practice

The results of the study demonstrate that malaria infection among children under 5 is an important public health problem in rural areas of the Dowa district. To address the problem, it is imperative that in addition to the available interventions such as LLINs and sulfadoxine-pyrimethamine preventive treatment programmes, health planners should also consider developing malaria control programmes that accommodate mothers without formal education. For example, the community-based, peer-to-peer, malaria education model has been an effective tool for behavioural changes in selected rural areas of southern Malawi, and may be applicable [42, 43].

The study also suggest that malaria control programmes in Dowa district should incorporate interventions that address IPV against mothers of young children. The current malaria proactive programmes in the Dowa district are gendered as they mainly target mothers by providing them with ITNs and by administering anti-malarial drugs during pregnancy. There is a need to involve fathers in all programmes that address child malaria. One such intervention could be a community-based participatory child malaria programmes that involve both men and women, as this has been found to improve fathers’ participation in childcare activities in similar contexts [44].

Finally, health professionals should consider engaging parents to find health promotion strategies that can reduce the risk of malaria among children 2 to 5 years old. The interventions should consider the developmental stages of children, geographical space, and the times of day and night that predispose these children to malarial vectors. For example, application of mosquito repellents can protect children from mosquito bites both indoors and outdoors. However, because current evidence on the effectiveness of repellents in the prevention of malaria in developing contexts is inconclusive, more research is needed before this intervention is adopted [45–47].

### Strengths and limitations of the study

The main strength of this study is that it is based on a systematic sampling technique and therefore, the findings can be generalized to all children 2 to 59 months old who accessed primary health care services in the studied clinics. Nevertheless, this study has some limitations. First, the study was conducted during the dry season, a period with significantly fewer mosquito-breeding sites compared to the wet season. Therefore, the findings do not represent seasonal variations in malaria prevalence. A suggestion is made that a longitudinal study should be conducted in order to provide a broader picture of malaria infection prevalence and risk factors in the study area. This study also used a cross-sectional design and as such, no causal inference can be made regarding the identified determinants and child malaria infection. Despite these limitations, the study has identified potential risk factors for malaria infection among children under 5 in rural areas of the Dowa district that can inform local programmes.

## Conclusion

The current study shows that the prevalence of malaria infection among children aged 2 to 59 months in rural areas of Dowa district was at 35.4%, which is equivalent to the prevalence of the phenomenon at the national level in 2017. Apart from well-known risk factors of child malaria infection such as child age range (24 to 59 months) and lack of maternal formal education, the study identified that maternal exposure to IPV is also a risk factor. Therefore, apart from increasing the distribution of treated mosquito nets and malaria screening, child malaria programmes in Dowa should also consider addressing IPV against mothers.

## Data Availability

The study involved gathering sensitive data according to WHO standards. We documented mothers’ disclosures of violence by their current husbands. Due to the sensitivity of the study, the two ethics boards did not recommend sharing the law data publicly.

## Abbreviations

AOR: Adjusted odds ratios
COR: Crude odds ratios
*CI*: Confidence interval
IPV: Intimate partner violence
ITN: Insecticide-treated bed nets
NMCP: National Malaria Control Programme
NSO: National Statistics Office
RDT: Rapid diagnostic test
SRQ: Self-reporting questionnaire
SSA: Sub-Sahara Africa
US$: United States dollar
WHO: World Health Organization
